# Machine Learning of Dose-Volume Histogram Parameters Predicting Overall Survival in Patients with Cervical Cancer Treated with Definitive Radiotherapy

**DOI:** 10.1155/2022/2643376

**Published:** 2022-06-14

**Authors:** Zhiyuan Xu, Li Yang, Qin Liu, Hao Yu, Longhua Chen

**Affiliations:** ^1^Department of Radiation Oncology, Nanfang Hospital, Southern Medical University, Guangzhou 510515, Guangdong Province, China; ^2^Clinical Oncology Center, The University of Hong Kong—Shenzhen Hospital, Shenzhen 518053, Guangdong Province, China; ^3^Department of Pathology, Zhujiang Hospital, Southern Medical University, Guangzhou 510282, Guangdong Province, China; ^4^Chinese Academy of Sciences Shenzhen Institutes of Advanced Technology, Shenzhen 518055, Guangdong Province, China

## Abstract

**Purpose:**

To analyze the effects of dosimetric parameters and clinical characteristics on overall survival (OS) by machine learning algorithms.

**Methods and Materials:**

128 patients with cervical cancer were treated with definitive pelvic radiotherapy with or without chemotherapy followed by image-guided brachytherapy. The elastic-net models with integrating DVH parameters and baseline clinical factors, only DVH parameters and only baseline clinical factors were constructed in 5-folds cross-validations for 100 iteration bootstrapping, and then were compared using concordance index (C-index) criteria. Finally, the selected important factors were used to build multivariable Cox-pH models for OS and also shown in nomograms for clinical usage.

**Results:**

The median OS occurred was 25.78 months with 25 (19.53%) deaths. The elastic-net models integrating clinical and DVH factors had the best prediction performances (C-index 0.76 in the train set and C-index 0.74 in the test set). Three important factors were selected, including baseline hemoglobin level as the protective factor, primary tumor volume (GTV_P) volume, and body V5 as the risk factors. The final multivariable Cox-pH models were constructed using these important factors and had prediction performance (C-index: 0.78, 95%CI: 0.73–0.81).

**Conclusions:**

This is the first attempt to establish elastic-net models to study the contributions of DVH parameters for predicting OS in patients with cervical cancer. These results can facilitate individualized tailoring of radiation treatment in cervical cancer patients.

## 1. Introduction

Cervical cancer is the fourth most frequently diagnosed cancer and the fourth leading cause of cancer death in women, with an estimated 604,000 new cases and 342,000 deaths worldwide in 2020 [1]. Multidisciplinary management planning based on the tumor size and extension made by a multidisciplinary tumor board before the start of any treatment is recommended by European Society for Medical Oncology (ESMO) guideline of cervical cancer [2]. For International Federation of Gynecology and Obstetrics (FIGO) stage IA1 to IB1, surgery is the main treatment with adjuvant radiotherapy (RT) ± chemotherapy in case of risk factors, and for the FIGO stage IB2–IVA, concurrent chemoradiotherapy (CCRT) represents the standard [2]. Definitive RT ± chemotherapy can also be used for patients with the FIGO stage IVB with oligometastasis [3] or who are not candidates for hysterectomy. Neoadjuvant chemotherapy remains controversial for locally advanced cervical cancer [2]. With regard to immunotherapy, in addition to its applications in recurrent or metastatic cervical cancer [4, 5], ongoing trials are investigating the combination of immunotherapy with RT or CCRT in locally advanced cervical cancer [5, 6]. Despite modern advances in various treatment modalities, the mortality of cervical cancer still remains high, with 5-year overall survival (OS) of about 65% after CCRT [7]. Therefore, it is crucial to identify prognostic factors to tailor personalized management strategies for patients with different risk levels.

The FIGO staging system has been the most commonly used method to classify the prognosis of cervical cancer patients. The 5-year OS rates of FIGO stage I-IV cervical cancer were 83%–100%, 70%–80%, 42%, and 32%, respectively [8, 9]. Over the years, researchers had made a tremendous effort to identify other clinical prognostic factors for OS [7]. High body mass index (BMI>25) at the time of cancer diagnosis was found to be positively associated with 2-year and 5-year survival rates [10]. Biological parameters, including pretreatment levels of hemoglobin, leucocyte, and platelet, were identified as prognostic factors for locally advanced cervical cancer [11]. A previously developed nomograms with C-index of 0.713 identified several prognostic factors associated with OS, including squamous cell carcinoma antigen (SCC–Ag), BMI, tumor size, pelvic wall involvement, and para-aortic lymph node metastasis [12]. Concurrent chemotherapy (≥4 cycles) [13], monocyte [14], age [15], and performance status were also found to be prognostic for survival. Another nomogram showed tumor size, grading, and parametria status affected 5-year OS in locally advanced cervical cancer primarily treated with neoadjuvant chemotherapy followed by radical surgery [16]. Nevertheless, the abovementioned prognostic factors mainly describe clinical features other than radiotherapy parameters. Since radiotherapy forms the backbone of cervical cancer treatment, it is reasonable to presume that dose-volume histogram (DVH) parameters may have an impact on OS. Analysis of dose-effect relationship between DVH parameters and prognosis for cervical cancer patients suggested that 100%, 98%, and 90% of high-risk clinical target volume received radiotherapy dose (HR-CTV D100, HR-CTV D98, and HR-CTV90) were independent factors affecting OS [17]. Retrospective DVH analysis showed that the equivalent dose in 2 Gy (EQD2) of HR-CTV D90 was significant determinant of OS in patients with uterine cervical cancer [13]. In addition to target volume, the prognostic impact of DVH parameters of organs at risk (OARs) has been studied in a range of cancer types. Multivariate analyses showed that lung V20 (volume covered by radiation dose of ≥ 20 Gy) and lung V5 (volume covered by radiation dose of ≥ 5 Gy) were associated with OS in patients with esophageal cancer treated with neoadjuvant chemoradiotherapy when adjusting for surgical margin and pathological treatment response [18]. To the best of our knowledge, there are no studies on the effect of DVH parameters of both tumor and OARs on OS during external beam radiotherapy (EBRT) of cervical cancer.

Different approaches can be employed to identify clinical and dosimetric parameters that affect the patient's outcome. A multidimensional nomogram has been developed for predicting progression-free survival (PFS) in patients with locoregionally advanced nasopharyngeal carcinoma [19]. The random survival forest model identified D99 (the dose that covered 99% of the volume) as an important variable associated with survival of high-grade glioma [20]. Besides the random survival forest model, the elastic-net model, as a machine learning method, yields higher discriminative performance in (chemo) radiotherapy outcome than other studied classifiers [21]. Therefore, in this study, we employed an elastic-net model to determine the key clinical and DVH parameters in predicting survival outcome of cervical cancer patients.

## 2. Materials and Methods

### 2.1. Description of Cohorts

The local institutional ethics committee approved the study (reference number (2019) 049). All patients provided written informed consent for the use of personal medical records for academic purpose before treatment and consent form for this specific study was waived.

A cohort of patients diagnosed with cervical cancer in a single institute in China from January 2015 to February 2021 was selected for this study. All patients were treated with definitive radiotherapy. Eligible patients met the criteria: ≥18 years old; previously untreated, pathologically confirmed cervical carcinoma; stage IB-IVB using FIGO (2018) (only stage IVB with oligo-metastasis scheduled for radical chemoradiotherapy included); main treatment was EBRT with or without chemotherapy followed by image-guided brachytherapy. Key exclusion criteria were the following: small cell carcinoma of the cervix, acquired immune deficiency syndrome, concomitant secondary primary malignancies, radiotherapy in adjuvant or recurrent settings, or patients who did not complete planned radiotherapy. The last follow-up time was 30 April 2021.

### 2.2. Radiation Therapy

All patients received EBRT using RapidArc or three-dimensional conformal radiotherapy (3D-CRT) techniques. EBRT was delivered on a 6 MV linear accelerator. For RapidArc, GTV_P, and GTV_N were defined as primary gross tumor volume and locoregional pathological lymph nodes detected by physical examination, pelvis magnetic resonance imaging (MRI), or positron emission tomography (PET)/CT. PTV_4500 and PTV_5500 (planning target volume of pelvis and metastatic lymph node received prescribed dose of 45 Gy and 55 Gy): prescription dose was 45 Gy in 25 fractions to PTV_4500 with a simultaneous integrated boost of 55 Gy to PTV_5500. For 3D-CRT, two sequential phases were used (45 Gy/25 fractions to pelvis for phase I; FIGO IIIB 16 Gy/8 fractions, other stages 10 Gy/5 fractions boosting to pelvic wall for phase II). All EBRT was daily, 5 fractions per week. CT or MRI guided brachytherapy was performed 3–4 weeks after initiation of EBRT with a 192 Ir (iridium) high dose rate, once a week for a total of 4 times. The cumulative equivalent of >84 Gy (EQD2) for stage IB-IIIA and >90 Gy (EQD2) for ≥ stage IIIB were set for the cervical tumor. DVH parameters during EBRT were obtained from the Varian Eclipse treatment planning system (version 15.0).

### 2.3. Chemotherapy

Concurrent cisplatin at 40 mg/m2 was given weekly during EBRT. Carboplatin (area under the curve (AUC) = 2 mg/ml/min) weekly was used as an alternative if creatinine clearance ≤50 ml/min. In cases involving long radiotherapy waiting time, induction chemotherapy with paclitaxel plus carboplatin was given. Chemotherapy was not recommended to patients aged over 70 or FIGO stage IB1.

### 2.4. Follow Up

In the first 2 years of follow-up, all the patients had regular assessment every 3 months, then every 6 months in the third to fifth year, and yearly after the fifth year. OS was the time from the start of EBRT to the date of death from any cause or the last confirmed date of survival.

### 2.5. Univariate Analysis and Multivariable Analysis

Univariate Cox-pH analysis was conducted to generate hazard ratios (HRs) with confidence intervals (CIs) of each single risk factor's contribution for OS. The factors extracted by elastic-net models were applied to build the final multivariable Cox-pH model. The Concordance index (C-index) were then applied to show the performance of the final multivariable Cox-pH model. The final multivariable Cox-pH models for predicting OS were used to construct nomograms.

### 2.6. Elastic-Net Modeling

Elastic-net regression is a type of penalized regression [22, 23]. Elastic-net uses both L1 norm penalty and L2 norm penalty on the regression covariates, and uses a mixing parameter that defines the proportion (alpha parameter) of penalty applied to the covariates between both L1 and L2 norms. Taken together, the elastic-net regression method allows retention of correlated covariates, but also regularizes model predictors in a manner that allows for improved prediction performance.

Elastic-net models were constructed for the prediction of OS using a 5-folds cross-validation methodology in 100 iterations bootstrapping, to approximate the models' generalization abilities when lacking an external validation dataset [21, 24]. To determine the important features for OS by elastic-net models, we selected the best alpha and lambda in the elastic-net model by the criteria of C-index. The features with significant coefficient in elastic-net models and high selected frequencies in bootstrapping were selected as important factors.

### 2.7. Statistical Considerations

All continuous features were normalized in log10(*x* + 1). All statistical analyses were performed by *R* software (version 4.0.2, *R* Development Core Team, Vienna, Austria). The *R* package glmnet was used to implement elastic-net modeling. *P* value less than 0.05 was considered statistically significant.

## 3. Results

### 3.1. Patient Characteristics

A total of 128 patients were assessed as eligible for inclusion in this study.  Table 1 lists detailed characteristics of the study population. The median OS was 25.78 (interquartile range, IQR: 14.26–41.57) months with 25 (19.53%) deaths. The median age was 53 (IQR: 46–63) years. 78.91% patients were treated with RapidArc and the others were treated with 3D-CRT. 20.31% patients had induction chemotherapy before EBRT and 84.38% patients had concurrent chemotherapy during radiotherapy. Results of univariate Cox-pH analysis of clinical factors influencing OS were also summarized in Table 1. Patients with a higher BMI or baseline hemoglobin levels had longer OS (HR: 1.06*e* - 3, 95%CI: 1.75*e* - 6–0.65, *P* value = 0.04; HR: 8.96*e* - 4, 95%CI: 4.34*e* - 6–0.18, *P* value <0.01, respectively); while patients had poor survival with FIGO 2018 stage IV (HR: 5.82, 95%CI: 1.44–23.48, *P* value = 0.01).

### 3.2. DVH Parameters

In this study, 20 DVH features were extracted, including dmax, dmean, and volume of tumor targets (GTV_P, GTV_N, PTV_4500, and PTV_5500), and dmax, dmean, *V*5, *V*45, and volume of OARs (body and bones) (Table 2). As summarized in Table 2, the median dmean of GTV_P was 47.1 (IQR: 46.6–49.21) Gy and the median dmax was 53.2 (IQR: 48.6–57.8) Gy. The median dmean of the whole body was 12.11 (IQR: 10.67–13.9) Gy and of the bones was 29.22 (IQR: 27.84–31.88) Gy.

Univariate Cox-pH analysis results of DVH parameters for OS are also presented in Table 2. Patients with poor survival had significantly higher volume metrics of tumor (GTV_P volume HR: 9.8, 95%CI: 2.67–35.91, *P* value: <0.01; PTV_4500 volume HR: 181.2, 95%CI: 1.98–1.66*e* + 04, *P* value = 0.02; GTV_N dmean low vs. none HR: 2.69, 95%CI: 1.06–6.8, *P* value = 0.04). Furthermore, patients with poor survival had a higher total body dose (dmean HR: 544.54, 95%CI: 7.9–3.75*e* + 04, *P*-value = 3.53*e* - 3; V5 HR: 991, 95%CI: 8.97–1.1*e* + 05, *P* value = 4.06*e*-03; V45 HR: 11.34, 95%CI: 1.13–114.19, *P* value = 0.04).

**Figure 1:**
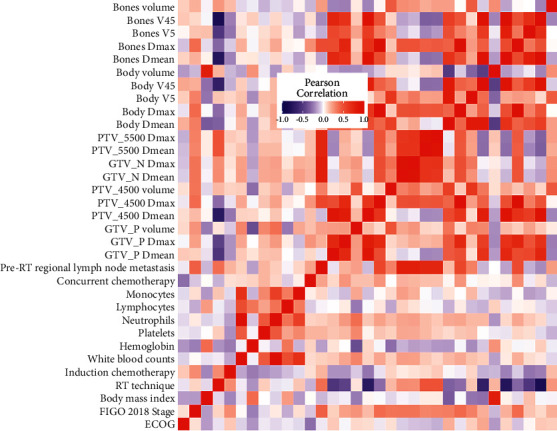
Pearson's correlations among clinical factors and DVH parameters. All continuous factors were normalized in log10 (*x* + 1).

Pearson's correlations between all DVH parameters are shown in Figure 1. Relatively strong positive correlations among the dosimetry of the tumor were found (Pearson's correlations > 0.5). While there were little correlations between clinical characteristics and DVH parameters, also little correlations among tumor dosimetry and OARs dosimetry.

### 3.3. Prediction Performances of Elastic-Net Models

**Figure 2:**
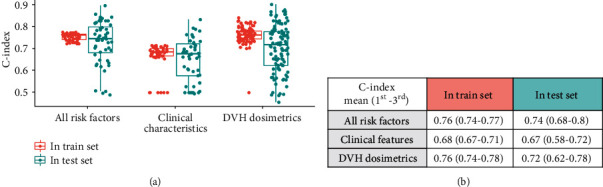
The performances of three kinds of elastic-net models summarized in the train set and the test set. The first model included both clinical features and DVH parameters, the second model included only clinical features, and the third model included only DVH parameters. Red represents C-index performances in the train set, and green represents C-index performances in the test set.

To study the risk factors of survival, three kinds of elastic-net models were established, including the model with integrating clinical factors and DVH parameters, with only clinical factors and with only DVH parameters. These three models had best prediction performances when alpha parameters equal to 0.8, 0.7, and 0.5, respectively. The prediction metric C-index was used to evaluate and compare the performances of three models in the train set and the test set as shown in Figure 2.

In the train set, models integrating clinical and DVH features had the best performances (C-index: 0.76, 1^st^–3^rd^ quartile: 0.74–0.77). Also, in test sets, the models integrating clinical and DVH features (C-index: 0.74, 1^st^–3^rd^ quartile: 0.68–0.8) performed much better than models based on clinical features only (C-index: 0.67, 1^st^–3^rd^ quartile: 0.58–0.72), and a little better than models with DVH parameters only (C-index: 0.72, 1^st^–3^rd^ quartile: 0.62–0.78). These results indicated DVH parameters had contributions to survival, future more indicated that DVH parameters applied complementary information of clinical factors in survival prediction.

### 3.4. Important Factors in Elastic-Net Models

**Figure 3:**
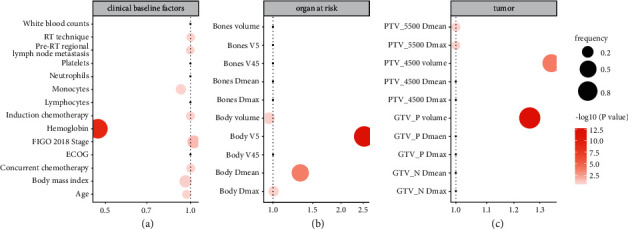
The important factors selected by elastic-net models including all factors, which constructed in 5-fold cross validation and 100 bootstrapping iterations. (a) Clinical factors; (b) DVH parameters of OARs; (c) DVH parameters of tumor. *X*-axis is the mean coefficient of one factor, color is its *P* value, and size is its frequency in 100 iterations.

The performances of all factors in the models with integrating clinical and DVH parameters were summarized, including the mean- and *P* value of their coefficients in the elastic-net models and the selected frequencies in 100 iterations, as shown in Figure 3 and (Supplemental Table 1). In clinical factors, the hemoglobin level at baseline was an important protective factor from death (mean coefficient: 0.47, 95%CI: 0.38–0.57, *P* value: <0.01, frequency:72%). In DVH parameters, both GTV_P volume and body *V*5 are the most promotive factors for death (mean coefficient: 1.26, 95%CI: 1.21–1.32, *P* value: <0.01, frequency: 92%; mean coefficient: 2.54, 95%CI: 2.1–3.09, *P* value: <0.01, frequency: 90%, respectively).

### 3.5. The Final Multivariable Cox-pH Model

**Figure 4:**
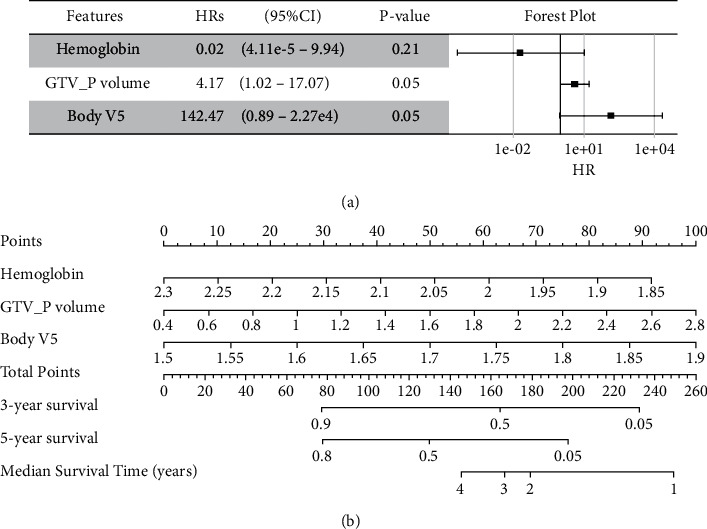
Multivariable Cox-pH models with the key factors selected by elastic-net models integrating clinical characteristics and DVH parameters. (a) The HRs (95%CI) and *P* value of risk factors in multivariable Cox-pH, and shown in the forest plot; (b) the corresponding nomogram. All continuous factors were normalized in log10 (*x* + 1).

For the possibility of clinical usage, the final multivariable Cox-pH model integrating the key clinical characteristics (hemoglobin at baseline) and DVH parameters (GTV_P volume and body *V*5) was constructed as shown in Figure 4(a) with C-index (0.78, 95%CI: 0.73–0.81). The corresponding nomogram for survival prediction were developed for clinical use as shown in Figure 4(b).

## 4. Discussion

The present study analyzed the effects of dosimetric parameters and clinical characteristics on OS by machine learning algorithms. The results showed that elastic-net models with integrating clinical and DVH factors had best prediction performances (C-index 0.76 in the train set and C-index 0.74 in the test set). Three important factors were selected, including baseline hemoglobin level, primary tumor volume (GTV_P), and body *V*5. The final multivariable Cox-pH model constructed using these important factors had prediction performance (C-index: 0.78, 95%CI: 0.73–0.81) better than previous studies [25–27]. It indicated that the addition of DVH parameters to clinical factors in the model improved the prediction ability for OS. At the same time, the final multivariable Cox-pH model and the nomogram plot with only three readily available indicators in practice making it feasible in clinical application.

In clinical factors, our study found that the hemoglobin level at baseline was an important protective factor from death which was widely acknowledged. Many other studies have reached similar conclusions. Pretreatment hemoglobin was found to be a potential biomarker for survival prognosis in not only early cervical cancer [28] but also locally advanced cervical carcinoma [12]. The first international expert consensus guideline informing a minimum hemoglobin transfusion target of 90 g/L was endorsed to balance tumor radiosensitivity with appropriate use of a scarce resource for patients with cervical cancer undergoing EBRT and brachytherapy [29]. The hemoglobin level more than 90 g/L at presentation was positively associated with a 5-year OS rate [30]. A new score identified <120 g/L for hemoglobin at the time of diagnosis impacted disease free survival (DFS) and OS [11].

In DVH parameters, both GTV_P volume and body V5 were the most promotive factors for death. It is consistent with the conclusions of other studies that the larger GTV_P volume, the worse the survival. The 5-year survival rate of cervical cancer patients with tumor volume <40 cm^3^ was significantly better than that of patients with >40 cm^3^ [31]. The total volume of metabolic tumors was an independent prognostic factor for the recurrence-free survival of patients undergoing radical radiotherapy and chemotherapy for cervical cancer [32]. Researches on other tumors also support this conclusion. GTV_P volume ≥5 cm^3^ was associated with a significantly worse OS in patients with sinonasal mucosal melanoma [33]. Another finding suggested that a pathological tumor volume of ≥18 cm^3^ was significantly correlated with shorter OS of oral squamous cell carcinoma [34]. Similar conclusion was also found in rectum cancer [35], nasopharyngeal carcinoma [36], supraglottic carcinoma [37], and glioblastoma [38].

Body *V*5 is, especially, an important risk DVH parameters we found for survival, which was little considered in radiation therapy before. There are two types of radiation health effect, including acute and late on-set disorders. Clinical symptoms of acute disorder begin with a decrease in lymphocytes, and then the symptoms appear, such as alopecia, skin erythema, hematopoietic damage, gastrointestinal damage, and central nervous system damage, with increasing radiation dose [39]. Body radiation can potentially result in both acute and long-lasting adverse effects, particularly, on hematopoietic and immune cells [40]. Studies have shown that radiation-induced lymphocytopenia is associated with poor prognosis in solid tumors [41], such as cervical cancer [42] and non-small cell lung cancer [43]. Regarding the late on-set disorder, predominant health effects are cancer [44–46], non-cancer disease [47, 48], and the genetic effect [49–51]. In addition, it should be noted that with the development of modern radiotherapy techniques, such as intensity-modulated radiotherapy (IMRT), patients receive a larger volume of low-dose radiation. Body dose-volume distributions may influence the risk of second primary cancer [52]. Moreover, radiation-induced normal tissue damage and repair also has a dose-volume effect [53].

There are some limitations in this study. First of all, this is a retrospective study. A prospective study is needed to collect more complete data. Secondly, since the international cervical cancer staging system does not include prognostic biomarkers, and current treatment recommendations are mainly based on staging, we did not include nonanatomical prognostic biomarkers, such as human papillomavirus (HPV) infection data and SCC-Ag values. Thirdly, the median follow-up for our analysis was 26.4 months, and longer follow-up is needed to fully assess long-term survival benefits. Lastly, although a 5-folds cross-validation methodology in 100 iterations bootstrapping was used to assure the models' generalization abilities, an external validation is needed in the future study. Nonetheless, the findings of our study provide valuable data to guide clinical practice and future research.

In conclusion, this is the first attempt to establish elastic-net models to evaluate the roles of DVH parameters in predicting OS in patients with cervical cancer. In addition to clinical factors, DVH parameters such as GTV_P volume and body *V*5 appear to be important predictors of survival outcome. These results can facilitate individualized tailoring of treatment and patient counseling in the holistic management of cervical cancer.

## Figures and Tables

**Table 1:** Summarization and univariate Cox-pH analysis of clinical factors for overall survival.

Features	Grades	Median (IQR) or No (%)	HR (95%CI)	*P* value
OS (months)		25.78 (14.26 – 41.57)		
Censor	0	103 (80.47%)		
	1	25 (19.53%)		
Age (years)		53 (46 – 63)	0.08 (9.08*e* − 04 – 6.87)	0.27
ECOG	0 and 1	112 (87.5%)	Reference	
	**2**	16 (12.5%)	1.74 (0.65 – 4.65)	0.27
FIGO 2018 stage	II	36 (28.12%)	Reference	
	III	83 (64.84%)	1.82 (0.61 – 5.43)	0.28
	IV	9 (7.03%)	5.82 (1.44 – 23.48)	0.01
Body mass index		23.05 (20.11 – 25.01)	1.06*e* − 03 (1.75*e* − 06 – 0.65)	0.04
EBRT technique	3D-CRT	27 (21.09%)	Reference	
	RapidArc	101 (78.91%)	0.9 (0.38 – 2.14)	0.81
Induction chemotherapy	Without	102 (79.69%)	Reference	
	With	26 (20.31%)	1.4 (0.52 – 3.8)	0.51
Concurrent chemotherapy	Without	20 (15.62%)	Reference	
	With	108 (84.38%)	0.7 (0.24 – 2.05)	0.52
Pre-RT regional lymph node metastasis	Without	43 (33.59%)	Reference	
	With	85 (66.41%)	1.48 (0.62 – 3.57)	0.38
White blood cells (10^3^ cells/ul)		6.59 (5.12 – 8.12)	1.85 (0.23 – 14.72)	0.56
Hemoglobin (g/L)		117.5 (102.5 – 129.25)	8.96*e* − 04 (4.34*e* − 06 – 0.18)	9.84*e*-03
Platelets (10^3^ cells/ul)		260 (217.5 – 318)	2.29 (0.17 – 30.44)	0.53
Neutrophils (10^3^ cells/ul)		4.24 (3.03 – 5.65)	2.61 (0.51 – 13.42)	0.25
Lymphocytes (10^3^ cells/ul)		1.74 (1.3 – 2.08)	0.11 (9.11*e* − 03 – 1.22)	0.07
Monocytes (10^3^ cells/ul)		0.34 (0.25 – 0.44)	0.34 (0.06 – 1.94)	0.22

Abbreviations: OS = overall survival; EBRT = external beam radiation therapy; 3D-CRT = three-dimensional conformal radiotherapy; RT = radiotherapy; ECOG = Eastern Cooperative Oncology Group; FIGO=International Federation of Gynecology and Obstetrics; IQR = interquartile range; HR = hazard ratio; CI = confidence interval.

**Table 2:** Summarization and univariate Cox-pH analysis of DVH parameters for overall survival.

Features	Grades	Median (1^st^ – 3^rd^) or Num (%)	HR (95% CI)	*P* value
GTV_P dmean (Gy)		47.1 (46.6 – 49.21)	1.26*e* + 04 (1.3*e* – 03 – 1.22*e* + 11)	0.25
GTV_P dmax (Gy)		53.2 (48.6 – 57.8)	5.93*e* + 03 (0.19 – 1.86*e* + 08)	0.1
GTV_P volume (cm^3^)		75 (52.18 – 122.58)	9.8 (2.67 – 35.91)	**5.77** *e*– **04**
PTV_4500 dmean (Gy)		47.62 (46.84 – 50.05)	6.68*e* + 03 (8.8*e* – 03 – 5.07*e* + 09)	0.2
PTV_4500 dmax (Gy)		58.5 (56.98 – 59.2)	9.2*e* + 03 (0.02 – 4.07*e* + 09)	0.17
PTV_4500 volume (cm^3^)		1.38*e* + 03 (1.25*e* + 03 – 1.54*e* + 03)	181.2 (1.98 – 1.66*e* + 04)	**0.02**
GTV_N dmean (Gy)	None	51 (39.84%)	Reference	
	Low	39 (30.47%)	2.69 (1.06 – 6.8)	**0.04**
	High	38 (29.69%)	1.12 (0.39 – 3.24)	0.83
GTV_N dmax (Gy)	None	51 (39.84%)	Reference	
	Low	42 (32.81%)	2 (0.76 – 5.27)	0.16
	High	35 (27.34%)	1.59 (0.59 – 4.24)	0.36
PTV_5500 dmean (Gy)	None	56 (43.75%)	Reference	
	Low	36 (28.12%)	1.81 (0.67 – 4.89)	0.24
	High	36 (28.12%)	1.37 (0.54 – 3.51)	0.51
PTV_5500 dmax (Gy)	None	56 (43.75%)	Reference	
	Low	37 (28.91%)	1.79 (0.66 – 4.84)	0.25
	High	35 (27.34%)	1.38 (0.54 – 3.53)	0.5
Body dmean (Gy)		12.11 (10.67 – 13.9)	544.54 (7.9 – 3.75*e* + 04)	**3.53** *e * **– 03**
Body dmax (Gy)		58.5 (56.98 – 59.23)	2.89*e* + 04 (0.05 – 1.54*e* + 10)	0.13
Body *V*5 (%)		45.32 (42.21 – 49.51)	991 (8.97 – 1.1*e* + 05)	**4.06** *e * **– 03**
Body *V*45 (%)		6.77 (5.37 – 9.47)	11.34 (1.13 – 114.19)	**0.04**
Body volume (cm^3^)		2.45*e*+04 (2.11*e* + 04 – 2.84*e* + 04)	0.02 (1.5*e*–04 – 2.4)	0.11
Bones dmean (Gy)		29.22 (27.84 – 31.88)	74.13 (0.15 – 3.58*e* + 04)	0.17
Bones dmax (Gy)		58.1 (53.88 – 58.8)	3.35*e* + 03 (0.01 – 1.04*e* + 09)	0.21
Bones V5 (%)		96.79 (95.69 – 98.5)	1.62*e* + 13 (1.29*e*-09 – 2.04*e* + 35)	0.24
Bones V45 (%)		13.77 (10.73 – 20.26)	3.12 (0.66 – 14.75)	0.15
Bones volume (cm^3^)		1.17*e* + 03 (1.07*e* + 03 – 1.28*e* + 03)	123.26 (0.12 – 1.23*e* + 05)>	0.17

dmax = maximum dose; dmean = mean dose; GTV_P or GTV_*N* = gross tumor volume of primary tumor or regionally metastatic lymph nodes, respectively; HR = hazard ratio; CI = confidence interval; PTV_4500 or PTV_5500 = planning target volume receiving prescription dose of 45 Gy or 55 Gy, respectively; *V*5 or *V*45 = the relative volumes (in percentage) covered by dose levels of ≥5 Gy or 45 Gy, respectively.

## Data Availability

The datasets analyzed during the current study are not publicly available because the data are strictly confidential, but are available from the corresponding author upon a reasonable request.

## Supplementary Materials

Supplementary Materials Supplemental Table 1 The factors in elastic-net models integrating clinical and dosimetric factors which were constructed in 5-fold cross validation and 100 bootstrapping iterations.

## References

[1] Sung H., Ferlay J., Siegel R. L. (2021). Global cancer statistics 2020: GLOBOCAN estimates of incidence and mortality worldwide for 36 cancers in 185 countries. *CA: A Cancer Journal for Clinicians*.

[2] Marth C., Landoni F., Mahner S., McCormack M., Gonzalez-Martin A., Colombo N. (2017). Cervical cancer: ESMO clinical practice guidelines for diagnosis, treatment and follow-up. *Annals of Oncology*.

[3] Chopra S., Mangaj A., Sharma A. (2021). Management of oligo-metastatic and oligo-recurrent cervical cancer: a pattern of care survey within the EMBRACE research network. *Radiotherapy and Oncology*.

[4] Colombo N., Dubot C., Lorusso D. (2021). Pembrolizumab for persistent, recurrent, or metastatic cervical cancer. *The New England Journal of Medicine*.

[5] De Felice F., Marchetti C., Palaia I. (2018). Immune check-point in cervical cancer. *Critical Reviews In Oncology-Hematology*.

[6] Feng C. H., Mell L. K., Sharabi A. B., McHale M., Mayadev J. S. (2020). Immunotherapy with radiotherapy and chemoradiotherapy for cervical cancer. *Seminars in Radiation Oncology*.

[7] Sturdza A., Pötter R., Fokdal L. U. (2016). Image guided brachytherapy in locally advanced cervical cancer: improved pelvic control and survival in RetroEMBRACE, a multicenter cohort study. *Radiotherapy and Oncology*.

[8] Tomizawa K., Kaminuma T., Murata K. (2020). FIGO 2018 staging for cervical cancer: influence on stage distribution and outcomes in the 3D-image-guided brachytherapy era. *Cancers*.

[9] Grigsby P. W., Massad L. S., Mutch D. G. (2020). FIGO 2018 staging criteria for cervical cancer: impact on stage migration and survival. *Gynecologic Oncology*.

[10] Huang X., Shu C., Chen L., Yao B. (2018). Impact of sex, body mass index and initial pathologic diagnosis age on the incidence and prognosis of different types of cancer. *Oncology Reports*.

[11] Maulard A., Chargari C., Faron M. (2020). A new score based on biomarker values to predict the prognosis of locally advanced cervical cancer. *Gynecologic Oncology*.

[12] Yang X., An J., Zhang Y. (2020). Prognostic nomograms predicting survival in patients with locally advanced cervical squamous cell carcinoma: the first nomogram compared with revised FIGO 2018 staging system. *Frontiers in Oncology*.

[13] Someya M., Hasegawa T., Tsuchiya T. (2020). Retrospective DVH analysis of point a based intracavitary brachytherapy for uterine cervical cancer. *Journal of Radiation Research*.

[14] Huang H., Liu Q., Zhu L. (2019). Prognostic value of preoperative systemic immune-inflammation index in patients with cervical cancer. *Scientific Reports*.

[15] Ryu J. M., Choi Y. S., Bae J. Y. (2020). Prognostic factors in women with cervical cancer stage IIIC1r treated with concurrent chemoradiotherapy. *Journal of Obstetrics and Gynaecology Research*.

[16] Marchetti C., De Felice F., Di Pinto A. (2018). Survival nomograms after curative neoadjuvant chemotherapy and radical surgery for stage IB2-IIIB cervical cancer. *Cancer Research and Treatment*.

[17] Zhang N., Tang Y., Guo X., Mao Z., Yang W., Cheng G. (2020). Analysis of dose-effect relationship between DVH parameters and clinical prognosis of definitive radio chemo therapy combined with intracavitary/interstitial brachytherapy in patients with locally advanced cervical cancer: a single-center retrospective study. *Brachytherapy*.

[18] Lin J.-B., Hung L.-C., Cheng C.-Y. (2019). Prognostic significance of lung radiation dose in patients with esophageal cancer treated with neoadjuvant chemoradiotherapy. *Radiation Oncology*.

[19] Yang K., Tian J., Zhang B. (2019). A multidimensional nomogram combining overall stage, dose volume histogram parameters and radiomics to predict progression-free survival in patients with locoregionally advanced nasopharyngeal carcinoma. *Oral Oncology*.

[20] Chen H., Li C., Zheng L., Lu W., Li Y., Wei Q. (2021). A machine learning-based survival prediction model of high grade glioma by integration of clinical and dose-volume histogram parameters. *Cancer Medicine*.

[21] Deist T. M., Dankers F. J. W. M., Valdes G. (2018). Machine learning algorithms for outcome prediction in chemo radiotherapy: an empirical comparison of classifiers. *Medical Physics*.

[22] Zou H., Hastie T. (2005). Regularization and variable selection via the elastic net. *Journal of the Royal Statistical Society: Series B*.

[23] Hastie T., Tibshirani R., Friedman J. (2009). *The Elements of statistical learning: data mining, inference, and prediction*.

[24] Xu C.-J., van der Schaaf A., Schilstra C., Langendijk J. A., Van’t Veld A. A. (2012). Impact of statistical learning methods on the predictive power of multivariate normal tissue complication probability models. *International Journal of Radiation Oncology, Biology, Physics*.

[25] Zang L., Chen Q., Zhang X. (2021). Nomogram predicting overall survival in patients with FIGO II to III squamous cell cervical carcinoma under radical radiotherapy: a retrospective analysis based on 2018 FIGO staging. *Cancer Management and Research*.

[26] Sturdza A. E., Pötter R., Kossmeier M. (2021). Nomogram predicting overall survival in patients with locally advanced cervical cancer treated with radiochemotherapy including image-guided brachytherapy: a retro-EMBRACE study. *International Journal of Radiation Oncology, Biology, Physics*.

[27] Kim J., Cho Y., Kim N. (2021). Magnetic resonance imaging-based validation of the 2018 FIGO staging system in patients treated with definitive radiotherapy for locally advanced cervix cancer. *Gynecologic Oncology*.

[28] Noh J. J., Lim M. C., Kim M. H. (2020). The prognostic model of pre-treatment complete blood count (CBC) for recurrence in early cervical cancer. *Journal of Clinical Medicine*.

[29] Zayed S., Nguyen T. K., Lin C. (2021). Red blood cell transfusion practices for patients with cervical cancer undergoing radiotherapy. *JAMA Network Open*.

[30] Khamis S. I., Mrema A. S., Katanga J., Lugina E. L. (2021). Survival in cervical cancer and its predictors at ocean road cancer institute from january to december 2012. *JCO Global Oncology*.

[31] Srivastava S., Saini S. K., Dixit A. K., Dwivedi D. (2017). Prognostic significance of tumor volume as determined on 3D ultrasound scan in uterine cervix cancer treated by radiotherapy. *Journal of Cancer Research and Therapeutics*.

[32] Hong J. H., Min K. J., Lee J. K. (2016). Prognostic value of the sum of metabolic tumor volume of primary tumor and lymph nodes using 18F-fdg PET/CT in patients with cervical cancer. *Medicine*.

[33] Flukes S., Lohia S., Barker C. A. (2020). Primary tumor volume as a predictor of distant metastases and survival in patients with sinonasal mucosal melanoma. *Head and Neck*.

[34] Mukoyama N., Suzuki H., Hanai N., Sone M., Hasegawa Y. (2018). Pathological tumor volume predicts survival outcomes in oral squamous cell carcinoma. *Oncology Letters*.

[35] Liu W., Li Y., Zhu H. (2021). The relationship between primary gross tumor volume and tumor response of locally advanced rectal cancer: pGTV as a more accurate tumor size indicator. *Journal of Investigative Surgery*.

[36] Qin L., Wu F., Lu H., Wei B., Li G., Wang R. (2016). Tumor volume predicts survival rate of advanced nasopharyngeal carcinoma treated with concurrent chemoradiotherapy. *Otolaryngology-Head and Neck Surgery*.

[37] Gong H., Zhou L., Hsueh C.-Y. (2021). Prognostic value of pathological tumor size in patients with supraglottic carcinoma. *American Journal of Otolaryngology*.

[38] Auer T. A., Della Seta M., Collettini F. (2021). Quantitative volumetric assessment of baseline enhancing tumor volume as an imaging biomarker predicts overall survival in patients with glioblastoma. *Acta Radiologica*.

[39] Kamiya K., Sasatani M. (2012). Effects of radiation exposure on human body. *Nihon Rinsho*.

[40] Hale L. P., Rajam G., Carlone G. M. (2019). Late effects of total body irradiation on hematopoietic recovery and immune function in rhesus macaques. *PLoS One*.

[41] Venkatesulu B. P., Mallick S., Lin S. H., Krishnan S. (2018). A systematic review of the influence of radiation-induced lymphopenia on survival outcomes in solid tumors. *Critical Reviews in Oncology-Hematology*.

[42] Onal C., Yildirim B. A., Guler O. C., Mertsoylu H. (2018). The utility of pretreatment and posttreatment lymphopenia in cervical squamous cell carcinoma patients treated with definitive chemoradiotherapy. *International Journal of Gynecological Cancer*.

[43] Bray F., Ferlay J., Soerjomataram I., Siegel R. L., Torre L. A., Jemal A. (2018). Global cancer statistics 2018: GLOBOCAN estimates of incidence and mortality worldwide for 36 cancers in 185 countries. *CA: a Cancer Journal for Clinicians*.

[44] Benjamin C., Shah J. K., Kondziolka D. (2020). Radiation-induced meningiomas. *Handbook of Clinical Neurology*.

[45] Ivanov V. K., Karpenko S. V., Kashcheev V. V. (2020). Relationship between follow-up periods and the low-dose ranges with statistically significant radiation-induced risk of all solid cancers in the Russian cohort of chernobyl emergency workers. *Radiation and Environmental Biophysics*.

[46] Sakata R., Preston D. L., Brenner A. V. (2019). Radiation-related risk of cancers of the upper digestive tract among Japanese atomic bomb survivors. *Radiation Research*.

[47] Park S., Lee D. N., Jin Y. W. (2021). Non-cancer disease prevalence and association with occupational radiation exposure among Korean radiation workers. *Scientific Reports*.

[48] Pasqual E., Boussin F., Bazyka D. (2021). Cognitive effects of low dose of ionizing radiation—lessons learned and research gaps from epidemiological and biological studies. *Environment International*.

[49] Repullés J., Anglada T., Soler D., Ramírez J. C., Genescà A., Terradas M. (2019). Radiation-induced malignant transformation of preneoplastic and normal breast primary epithelial cells. *Molecular Cancer Research: MCR*.

[50] Shemetun O., Pilinska M. A., Pilinska M. (2019). Radiation-induced bystander effect - modeling, manifestation, mechanisms, persistence, cancer risks literature review. *Problems of Radiation Medicine and Radiobiology*.

[51] Guéguen Y., Bontemps A., Ebrahimian T. G. (2019). Adaptive responses to low doses of radiation or chemicals: their cellular and molecular mechanisms. *Cellular and Molecular Life Sciences: CM*.

[52] Journy N., Mansouri I., Allodji R. S. (2019). Volume effects of radiotherapy on the risk of second primary cancers: a systematic review of clinical and epidemiological studies. *Radiotherapy and Oncology*.

[53] Bahn E., van Heerden M., Sachse K. N. (2020). Volume-dependent dose-response of the intestinal stem cell niche and lymphoid tissue. *Radiotherapy and Oncology*.

