# Supplementary material for: Machine Learning of Dose-Volume Histogram Parameters Predicting Overall Survival in Patients with Cervical Cancer Treated with Definitive Radiotherapy
Source: J Oncol. 2022 Jun 14;2022:2643376. doi: 10.1155/2022/2643376 (PMC9213181; doi:10.1155/2022/2643376)
Supplement: Supplementary Materials — Supplemental Table 1 The factors in elastic-net models integrating clinical and dosimetric factors which were constructed in 5-fold cross validation and 100 bootstrapping iterations. [file 2643376.f1.doc]

| Supplemental Table 1. The factors in elastic-net models integrating clinical and dosimetrics factors which were constructed in 5-fold cross validation and 100 bootstrapping iterations. | | | | | |
| --- | --- | --- | --- | --- | --- |
| Factors | Frequency | | Coef | (95%CI) | P-value |
| Age | 0.02 | 0.97 | | (0.92 - 1.03) | 0.32 |
| ECOG | 0 | 1 | | (/ - /) | / |
| FIGO 2018 stage | 0.1 | 1.03 | | (1 - 1.05) | 0.03 |
| Body mass index | 0.08 | 0.96 | | (0.92 - 1.01) | 0.14 |
| RT technique | 0.02 | 1 | | (1 - 1) | 0.32 |
| Induction chemotherapy | 0.02 | 1 | | (1 - 1.01) | 0.32 |
| White blood cells | 0 | 1 | | (/ - /) | / |
| Hemoglobin | 0.72 | 0.47 | | (0.38 - 0.57) | 9.08E-10 |
| Platelets | 0 | 1 | | (/ - /) | / |
| Neutrophils | 0 | 1 | | (/ - /) | / |
| Lymphocytes | 0 | 1 | | (/ - /) | / |
| Monocytes | 0.04 | 0.93 | | (0.82 - 1.05) | 0.24 |
| Concurrent chemotherapy | 0.02 | 1 | | (0.99 - 1) | 0.32 |
| Pre-RT regional lymph node metastasis | 0.02 | 1 | | (1 - 1) | 0.32 |
| Body Dmean | 0.44 | 1.32 | | (1.15 - 1.51) | 1.74E-04 |
| Body Dmax | 0.04 | 1.01 | | (0.99 - 1.03) | 0.23 |
| Body V5 | 0.9 | 2.54 | | (2.1 - 3.09) | 5.82E-13 |
| Body V45 | 0 | 1 | | (/ - /) | / |
| Body volume | 0.08 | 0.96 | | (0.91 - 1.01) | 0.11 |
| Bones Dmean | 0 | 1 | | (/ - /) | / |
| Bones Dmax | 0 | 1 | | (/ - /) | / |
| Bones V5 | 0 | 1 | | (/ - /) | / |
| Bones V45 | 0 | 1 | | (/ - /) | / |
| Bones volume | 0 | 1 | | (/ - /) | / |
| GTV_P Dmean | 0 | 1 | | (/ - /) | / |
| GTV_P Dmax | 0 | 1 | | (/ - /) | / |
| GTV_P volume | 0.92 | 1.26 | | (1.21 - 1.32) | 1.73e-13 |
| PTV_4500 Dmean | 0 | 1 | | (/ - /) | / |
| PTV_4500 Dmax | 0 | 1 | | (/ - /) | / |
| PTV_4500 volume | 0.48 | 1.35 | | (1.17 - 1.56) | 1.32e-04 |
| GTV_N Dmean | 0 | 1 | | (/ - /) | / |
| GTV_N Dmax | 0 | 1 | | (/ - /) | / |
| PTV_5500 Dmean | 0.02 | 1 | | (1 - 1) | 0.32 |
| PTV_5500 Dmax | 0.02 | 1 | | (1 - 1) | 0.32 |

Coef=coefficiency; CI=confidence interval; ECOG=Eastern Cooperative Oncology Group; FIGO, International Federation of Gynecology and Obstetrics; RT=radiotherapy; Dmax=maximum dose; Dmean=mean dose; GTV_P or GTV_N =gross tumor volume of primary tumor or regionally metastatic lymph nodes, respectively; PTV_4500 or PTV_5500=planning target volume receiving prescription dose of 45Gy or 55Gy, respectively; V5 or V45=the relative volumes (in percentage) covered by dose levels of ≥ 5Gy or 45Gy , respectively.
